# Safety of a novel oral immunotherapy approach in preschool children with single and multiple food allergies

**DOI:** 10.3389/falgy.2026.1724759

**Published:** 2026-02-02

**Authors:** Lieke J. C. Barten, Joyce Faber, Kim Kamphorst, Daphne Philips, Johan Garssen, Ted Klok

**Affiliations:** 1 Pediatric Allergy Treatment Center, Deventer Hospital, Deventer, Netherlands; 2 Utrecht Institute for Pharmaceutical Sciences, Division Pharmacology, Utrecht University, Utrecht, Netherlands

**Keywords:** allergen immunotherapy, children, clinical trial, dosing reactions, food allergy, oral immunotherapy, preschoolers, safety

## Abstract

**Background:**

Oral immunotherapy (OIT) has emerged as a promising food allergy treatment with significant evidence of increased efficacy in preschool-aged children. However, current OIT protocols are generally burdensome and focus on single food allergies, while many children have multiple food allergies. A feasibility-oriented approach that minimizes the impact of therapy while allowing both single and multiple food allergy treatment is important to enhance accessibility for routine clinical practice. This study assesses the safety of a feasibility-oriented OIT protocol in preschool-aged children with single and multiple food allergies.

**Methods:**

This single-center prospective intervention study included children aged 9–24 months with proven food allergy (sensitization and a positive oral food challenge (OFC)). After a short dose escalation phase, children received one year of low-dose (300 mg protein/day) OIT, followed by an exit OFC four weeks after stopping therapy. Children with multiple food allergies received OIT for up to four allergens. Each allergen treatment is referred to as an OIT trajectory. Allergic dosing reactions (ADR) were recorded and classified using Sampson's severity score (grades I-V).

**Results:**

Between May 2019 and Oct 2022, 124 children (median age 17 months, IQR 11–20 months) started OIT for 189 food allergies. Peanut (*n* = 52), cashew nut (*n* = 46) and egg (*n* = 38) allergies were the most common. The median number of hospital-based dose escalations per patient was three (IQR 2–5). ADR occurred in 89 children and 117 OIT trajectories, most frequently during dose escalation (48.1%). Most reactions (95.3%) were mild (Sampson I-II) and resolved spontaneously (55.6%) or with antihistamines (29.9%). Ten reactions were severe (Sampson III-IV), of which one required epinephrine. Ten trajectories were discontinued due to side effects.

**Conclusions:**

Our novel feasibility-oriented OIT protocol appears safe for various allergens and multifood OIT in preschool-aged children. Side effects were common but typically mild. However, they may lead to therapy discontinuation.

**Clinical Trial Registration:**

https://www.onderzoekmetmensen.nl/en/trial/49735, identifier NL7663.

## Highlights

▪ Preschool oral immunotherapy using a feasibility-oriented protocol appears safe for various allergens and children with multiple food allergy
▪ Allergic dosing reactions are common, but usually mild and self-limiting; epinephrine is rarely needed
▪ A family-friendly oral immunotherapy protocol as used in this study may greatly facilitate implementation of preschool oral immunotherapy in daily practice

## Introduction

Food allergies typically develop early in life in infants with atopic dermatitis (1–6). Currently, parents of atopic children are advised to introduce (potentially) allergenic foods early in life to reduce the risk of developing a food allergy (1, 7, 8). For those who develop food allergy despite these efforts, the standard of care is strict dietary avoidance and rescue medication. However, there is a shift to more active management with individual dietary advice (e.g., introduction of baked foods in children with milk/egg allergies) and oral immunotherapy (OIT) as immunomodulatory intervention (9). This shift is based on findings that the immune response of young children is more plastic and more amenable to immunomodulation, which may confer a critical early life window of opportunity to induce tolerance (10).

Most studies investigating OIT have focused on school-aged children and adults. These studies showed that OIT can induce desensitization; the ability to consume a specified amount of the food without dose-limiting symptoms while the patient is on therapy (9, 11, 12). Studies of preschool peanut OIT have shown efficacy in terms of sustained unresponsiveness development; the ability to consume a specified amount of the food without dose-limiting symptoms after stopping OIT (13–15). This highly desirable outcome was seen in over 70% of preschool-aged children. For allergens other than peanut, research on the induction of sustained unresponsiveness is lacking. A few studies on tree nut, sesame and fresh cow's milk OIT show promise regarding its safety for these allergens in preschool-aged children (13, 15–20). If the enhanced efficacy of peanut OIT in preschool-aged children compared to school-aged children and adults is also true for other allergens, then OIT can be expected to become a new standard of care for preschool-aged children with food allergies.

Envisioning OIT as standard of care, the impact of OIT on allergy health practice and families of food-allergic children will depend on how the therapy is executed. In general, OIT regimes are burdensome, with long dose escalation phases and multiple hospital visits (10). In addition, patients frequently have multiple food allergies where it is preferable to offer multifood OIT, but this presents additional challenges. To limit the impact of therapy, we designed a feasibility-oriented OIT protocol aimed to be suitable for routine clinical practice, targeting both single and multifood therapy for various allergens. In this paper, we describe the safety of this approach as a critical first step in a cohort of preschool-aged children with mild to severe food allergies.

## Materials and methods

### Study design

The ORKA study is a single-center explorative prospective intervention study conducted at the pediatric allergy treatment center of the Deventer Hospital in the Netherlands. The Deventer Hospital is a community hospital with supra-regional pediatric allergy care and a member of the collaborating top clinical teaching hospitals in the Netherlands. Ethics approval was granted by Medical Research Ethics Committee Isala clinics Zwolle, the Netherlands on 21-01-2019 (approval number 181021). The study was monitored by a Drug and Safety Monitoring Board. The trial is registered in the Dutch Trial Register (NL7663) (21). Participants were enrolled between May 2019 and October 2022. Written informed consent was obtained from parents/guardians.

### Study population

Patients were enrolled in the outpatient allergy clinic, where they were either already under treatment or to which they were referred from primary care and hospitals across the country to be screened for participation. Children aged 9–24 months were eligible for inclusion if they fulfilled the following criteria: a proven food allergy based on (1) a positive skin prick test (SPT) result (wheal diameter > 3 mm) and/or allergen-specific IgE (sIgE) level > 0.35 kU/L; and (2) a positive clinical oral food challenge (OFC) no later than three months before the start of OIT. Open OFCs were performed according to the PRACTALL Consensus Report (a semilogarithmic 8-step scale of 1–3,000 mg allergen protein, ending with an age-appropriate serving) (22). OFC outcomes were determined using pre-set criteria based on the PRACTALL Consensus Report (Supplementary Material 2c).

Children were excluded if they had severe gastrointestinal symptoms such as gastroesophageal reflux disease in which Eosinophilic Esophagitis (EoE) has not been ruled out, active EoE, mastocytosis, uncontrolled atopic dermatitis, or uncontrolled wheezing, defined as >1 hospitalization for these symptoms in the past six months. Contraindications for inclusion at the allergist's discretion were psychosocial problems in the family that may interfere with proper daily implementation of therapy, and parental inability to follow instructions, recognize allergic reactions or administer emergency medication.

### OIT protocol development

Together with parents of food-allergic preschool-aged children, a feasibility-oriented OIT protocol was developed. In designing the protocol, important goals were to minimize the impact of therapy, optimally use the window of opportunity to induce tolerance in early life, and normalize dietary habits as quickly as possible. From the start, the protocol was based on low-dose OIT (300 mg daily dose) during one year, a personalized starting dose and the use of commonly available food products.

The OFC before inclusion in the study determined the allergic reaction threshold, defined as the step (measured in milligrams of allergenic protein) at which objective symptoms occurred. Children started OIT with an initial dose of 30% of the allergic reaction threshold, with a maximum of 300 mg allergen protein.

Children with a threshold level ≥1,000 mg allergen protein directly started with an in-hospital ingestion of the maintenance dose of 300 mg allergen protein. Children with a threshold level <1,000 mg allergen protein received a personalized schedule of in-hospital dose escalations performed every 2–4 weeks to reach the maintenance dose. Between clinic visits, children ingested the specific food daily at home. OIT was performed with commonly available food products that parents purchased themselves, such as peanut butter, semi-skimmed cow's milk, boiled egg, and ground unroasted and unsalted nuts. Parents received a tested precision scale and instructions to weigh the daily dose themselves at home, except for doses under 0.3 g of food product, which were weighed at the hospital with a validated 1-mg precision scale.

Maintenance therapy lasted for one year, followed by a four-week period of strict food allergen avoidance. After this period, an exit OFC was performed to assess sustained unresponsiveness (according to the PRACTALL Consensus Report, see Supplementary Information). If children passed this challenge, they were advised to introduce the allergen into their diet with a minimum weekly intake of a full child's serving.

Because of the limited evidence on the safety of multifood OIT, the first group of participants followed this basic protocol which prioritized safety by limiting each participant to single OIT. In case of multiple food allergies, participants received consecutive treatments for their allergies. In addition, the basic protocol used a dose escalation schedule with one escalation per hospital visit (regular schedule).

**Figure 1:**
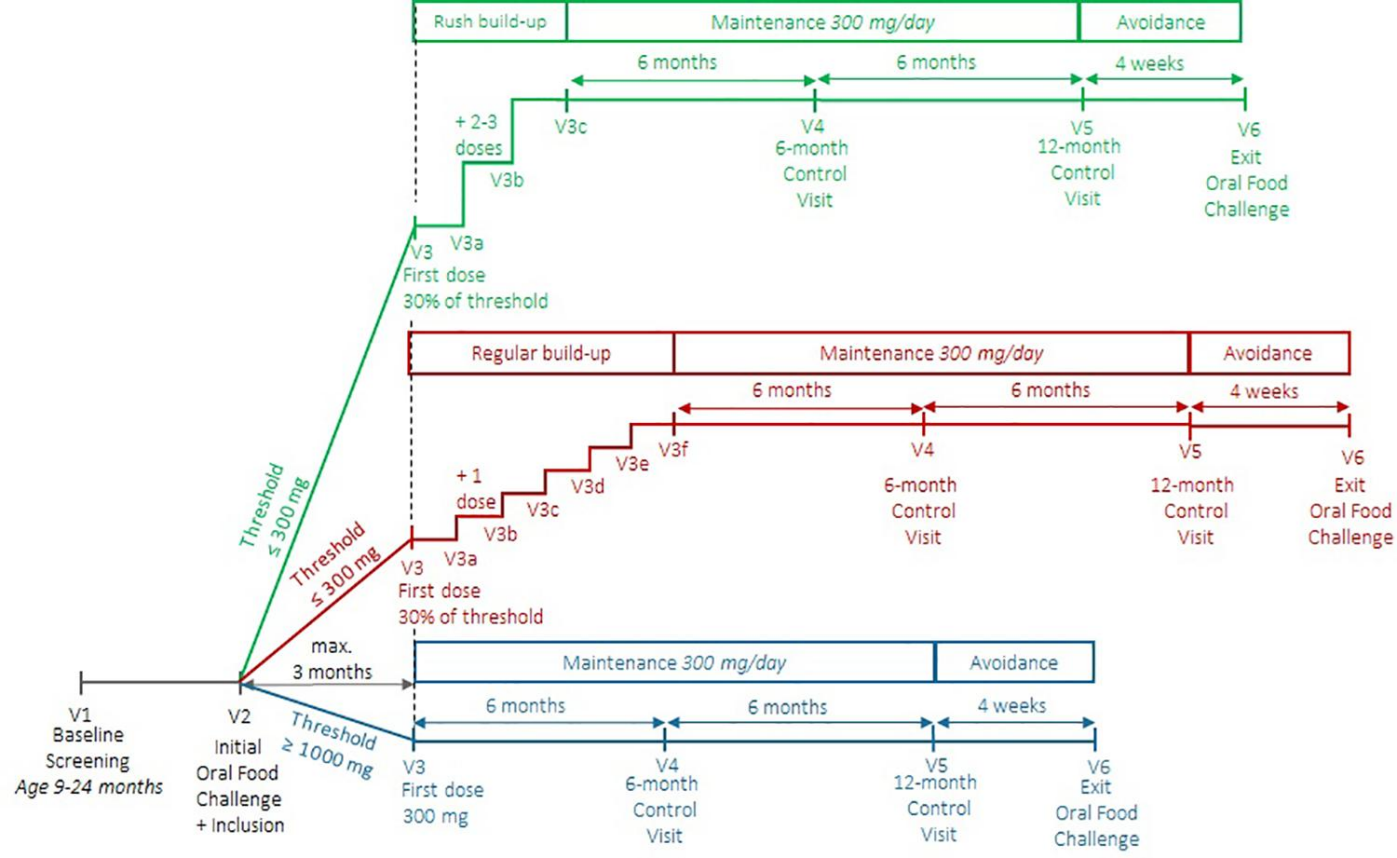
Children were screened for participation at the age of 9–24 months. During the initial oral food challenge, their baseline allergic reaction threshold was determined. OIT was started within 3 months of the initial oral food challenge. Children with a baseline threshold ≥1,000 mg protein could directly start with the maintenance dose of 300 mg protein/day. Children with a baseline threshold ≤300 mg protein started with a first dose of 30% of their threshold, followed by a build-up phase to reach the maintenance dose. For build-up, they could chose between a regular schedule (1 dose/visit) or a rush schedule (2–3 doses/visit). Children with multiple food allergies built up on the same days, with a maximum total of 6 doses/visit. The maintenance phase of 300 mg protein/day lasted 12 months, followed by a 4-weeks stop period and then an exit oral food challenge to assess sustained unresponsiveness.

To further minimize the impact of OIT, simultaneous treatment of multiple food allergies (multifood OIT), and a dose escalation schedule with two to three dose escalations per hospital visit (rush schedule) were added as options as of July 2020, inclusion number 36. Parents and caregivers of this second group of children together chose for either the regular or rush schedule (see Supplementary Information). Children with multiple food allergies received OIT for all allergens eligible for inclusion, with a maximum of four. For those with multiple food allergies, dose escalations were performed on the same days, with a maximum total of six dose escalations per day. An overview of the final study procedures is presented in Figure 1.

(Severe) adverse events [(S)AE] and their management during clinical dose escalation and between visits were recorded in the patient's medical chart and the electronic study database. All patients had antihistamine and an epinephrine auto injector available at home as rescue medication. Study participants received a standardized flow sheet with instructions on how to manage allergic reactions and when to halve or hold OIT doses (e.g., during viral illness) (see Supplementary Information).

### Outcomes

Primary outcomes included the occurrence and severity of allergic reactions within two hours of OIT administration, treatment of allergic reactions (including need for epinephrine), need for an emergency department visit, measures taken regarding adjustments of OIT, and dropout during the dose escalation or maintenance-dosing phase. The severity of OIT dosing reactions was assessed using Sampson's severity score, a widely used scoring system in the Netherlands (23). The scoring includes 5 grades from mild (Sampson I and II) to severe (Sampson III-V) allergic reactions. We defined localized allergic reactions as Sampson I and systemic reactions as Sampson II-V. Subgroup analyses were performed for trajectories with and without a dose escalation phase (low threshold <1,000 mg vs. high threshold ≥1,000 mg) and for different allergens. Differences in severity of allergic reactions were studied for treatment characteristics such as regular vs. rush dose escalation, and single vs. multifood OIT.

Secondary safety outcomes included the incidence of other symptoms/complaints (e.g., abdominal pain or allergic reactions > 2 h after OIT administration) or specific diseases (e.g., EoE) that may be related to OIT.

### Statistical analysis

Outcomes are presented as numbers and percentage of patients and/or OIT trajectories. We defined an OIT trajectory as one allergen treated with OIT. For example, if a patient undergoes OIT for three allergens, this patient is on three OIT trajectories. We defined multifood OIT as the simultaneous administration of OIT for at least two distinct food allergies in one patient.

Descriptive statistics were used to describe the characteristics of the study population, and the safety outcomes. Continuous variables were described as mean values with standard deviation (SD) if normally distributed and median values with interquartile range (IQR) if not normally distributed. Categorical variables were described as frequencies with percentages. Differences between (sub)groups were also presented descriptively. Categorical variables were compared using Chi-squared tests or Fisher's exact tests if applicable. Continuous variables were compared using unpaired student's *t*-tests for normally distributed data and Mann–Whitney *U*-tests for not normally distributed data. Statistical significance was set at *P* ≤ .05. Given the exploratory study design, small subgroup sizes, and high number of clinical parameters, subgroup analyses were presented descriptively without formal statistical testing to avoid overinterpretation. Data were analyzed using IBM SPSS Statistics for Windows, Version 29.0.2.0 Armonk, NY: IBM Corp (Released 2023).

## Results

### Baseline characteristics

**Figure 2:**
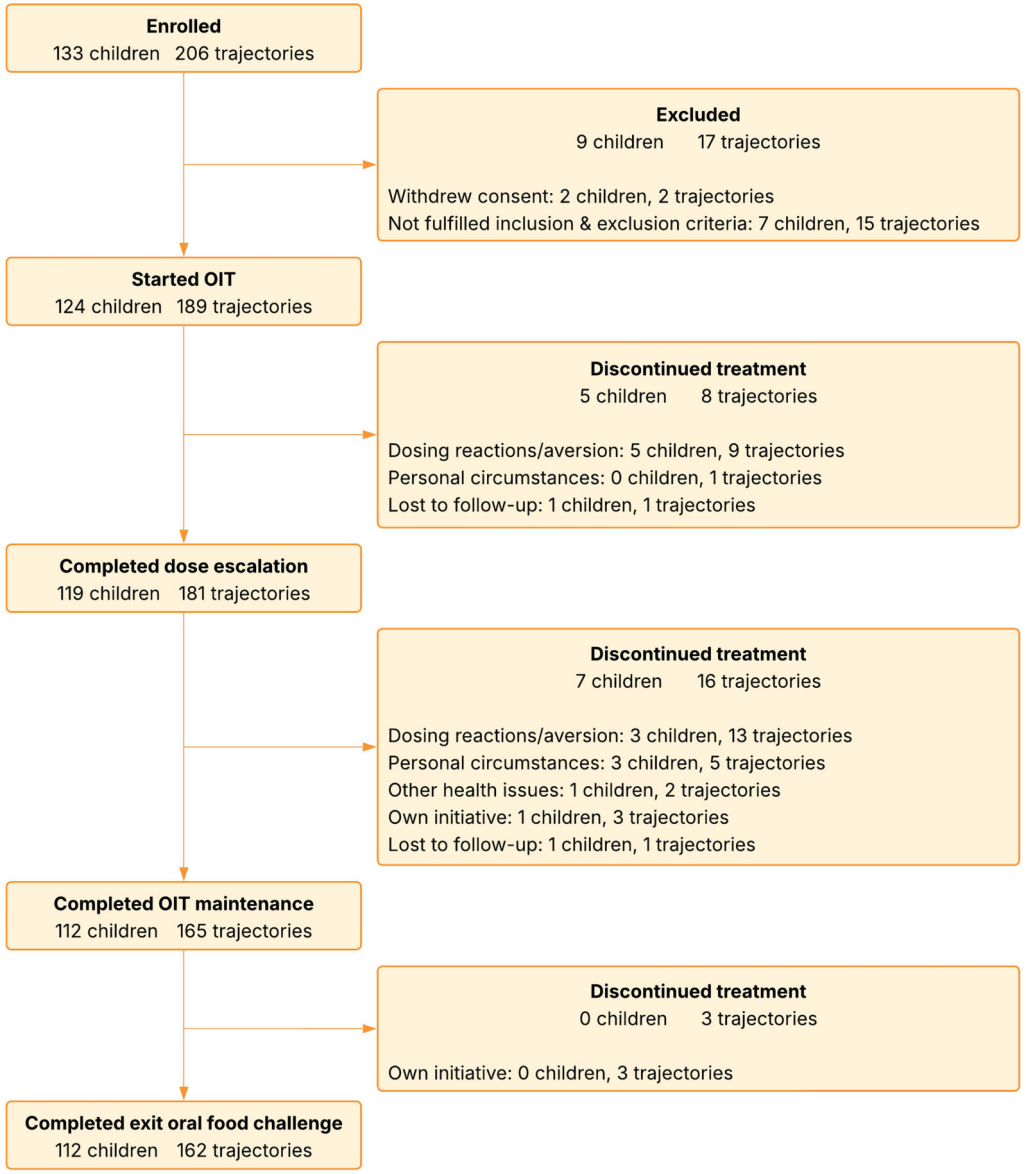
Children were screened for participation according to the study protocol to start OIT for all proven IgE-mediated food allergies, up to a maximum of four per child. After the entry oral food challenge**(s)** to 4.4 g of allergen protein, participants consumed daily OIT for 12 months, followed by a 4-week stop period with strict allergen avoidance and then an exit oral food challenge to 4.4 g of allergen protein to assess for sustained unresponsiveness. † Trajectories: an individual food allergen (to be) treated with OIT. ‡ More than one option can be applicable. # Multifood OIT: simultaneous OIT for at least two distinct food allergies in one child, with the treatment trajectories intersecting in time. OIT, oral immunotherapy.

Between May 2019 and October 2022, 124 children were enrolled in this study (Figure 2). Their median age was 17 (IQR 11–20) months at inclusion and 20 (IQR 14–23) months at the start of OIT (Table 1). The median severity of the food allergic reaction at the baseline OFC, was Sampson II (range II–V). Twenty-seven (21.8%) participants had a history of anaphylaxis (Sampson IV–V) for at least one of the allergens for which they received OIT. The median reaction threshold at the baseline OFC was 300 mg of allergen protein (IQR 300–1,000 mg). The participants together received 189 OIT trajectories; 80 participants received single OIT and 44 received multifood OIT. The most frequently treated allergens were treated were peanut (*n* = 52), cashew nut (*n* = 46) and hen's egg (*n* = 38). In total, 100 OIT trajectories needed dose escalation (baseline threshold ≤300 mg) and 88 trajectories could directly start with the maintenance dose (baseline threshold ≥1,000 mg). See Supplementary Table S1 for the split baseline characteristics of these two groups. The median number of hospitalizations for dose escalations was three (IQR 2–5). A rush dose escalation schedule was used in 86 OIT trajectories (86.0%).

**Table 1:** Baseline characteristics.

Characteristic	Total *n* = 124, *t* = 189
Age (mo) at inclusion, median (IQR)	17 (11–20)
Age (mo) at start of OIT, median (IQR)	20 (14–23)
Sex, *n* (%)	
Male	85 (68.5)
Female	39 (31.5)
Grade of allergic reaction at entrance OFC^a^, t (%)	
Grade II	152 (80.4)
Grade III	18 (9.5)
Grade IV	17 (9.0)
Grade V	2 (1.1)
Median grade (IQR)	2 (2–2)
History of anaphylaxis (grade IV or V^a^), t (%)	27 (21.8)
Threshold level of the allergic reaction at the entrance OFC, t (%)	
1 mg allergenic protein	1 (0.5)
3 mg allergenic protein	2 (1.1)
10 mg allergenic protein	9 (4.8)
30 mg allergenic protein	7 (3.7)
100 mg allergenic protein	25 (13.2)
300 mg allergenic protein	56 (29.6)
1,000 mg allergenic protein	51 (27.0)
3,000 mg allergenic protein	37 (19.6)
Missing	1 (0.5)
Median threshold level (IQR)	300 (300–1,000)
Baseline sIgE (kU/L), median (IQR)	
Peanut	7.0 (2.5–16.3)
Ara H2 (peanut)	4.5 (1.8–12)
Hen's egg	6.3 (2.6–19)
Cow's milk	11 (2.8–73)
Hazelnut	16 (5–24)
Cor A9 (hazelnut)	2.5 (1.1–4.2)
Cor A14 (hazelnut)	6.3 (0.5–29.2)
Cashew nut	9.4 (3.4–23.9)
Walnut	10.1 (4.9–18.1)
History of atopic dermatitis *n* (%)	119 (96)
History of asthma/viral wheezing, *n* (%)	24 (19.4)
No. of food allergies treated with OIT, *n* (%)	
1 food allergen	74 (59.7)
2 food allergens	36 (29)
3 food allergens	13 (10.5)
4 food allergens	1 (0.8)
Single or multifood OIT: single OIT^c^, *n* (%)	80 (64.5)
No. of dose escalations^b^, median (IQR)	3 (2–5)
Regular or rush dose escalation schedule: rush, t (%)	86 (86)
Food allergen treated with OIT, *n* (%)	
Peanut	52 (27.5)
Hen's egg	38 (20.1)
Cow's milk	7 (3.7)
Hazelnut	21 (11.1)
Cashew nut	46 (24.3)
Walnut	16 (8.5)
Almond	2 (1.1)
Pine nut	2 (1.1)
Sesame	3 (1.6)
Lentil	1 (0.5)
Wheat	1 (0.5)

OIT, oral immunotherapy; OFC, Oral Food Challenge; t, OIT trajectories; mo, months; IQR, interquartile range.

a Allergic reactions were classified using Sampson's Severity Score.

b For patients with a threshold ≤300 mg.

c Multifood OIT was defined as the simultaneous administration of OIT for at least two distinct food allergies in one patient, with the treatment trajectories intersecting in time.

### Allergic dosing reactions

Dosing reactions within two hours of OIT ingestion occurred in all study phases and in 89 (71.8%) of 124 participants (Table 2). Of these, nine participants (7.3%) had ≥five reactions. Dosing reactions were most frequent during the dose escalation phase. Overall, dosing reactions occurred in 111 (58.7%) of 189 OIT trajectories. Approximately half of the dosing reactions were systemic allergic reactions, including 10 (4.7%) severe reactions. The overall most frequently reported symptoms were mild rhinitis, oral itching, and local skin reactions. The ten severe reactions occurred during clinical dose escalation in six participants (three repeated vomiting, one wheezing, and two hoarseness) and at-home maintenance dosing in four participants (two repeated vomiting and two hoarseness).

**Table 2:** Allergic dosing reactions.

Characteristic	Total *n* = 124, *t* = 189
No. of patients with (an) allergic reaction(s), *n* (%)	89 (71.8)
No. of patients with ≥5 allergic reactions, *n* (%)	9 (7.3)
No. of OIT trajectories with (an) allergic reaction(s), t (%)	111 (58.7)
No. of allergic reactions reported, no. (%)	
Total	214 (100)
Dose escalation phase; first dose	36 (16.8)
Dose escalation phase; remaining	103 (48.1)
Maintenance phase; 0–4 mo	37 (17.3)
Maintenance phase; 4–8 mo	21 (9.8)
Maintenance phase; 8–12 mo	17 (7.9)
Severity^a^, no. (%)	
Grade I	104 (48.6)
Grade II	100 (46.7)
Grade III	5 (2.3)
Grade IV	5 (2.3)
Grade V	0 (0)
Organ system involved^b^, no. (%)	
Lower airways	1 (0.5)
Upper airways, mild/moderate	47 (22.0)
Upper airways, severe	4 (1.9)
Cardiovascular system	0 (0)
Skin/mucosa, mild/moderate	123 (57.5)
Skin/mucosa, severe	37 (17.3)
Gastro-intestinal, mild/moderate	66 (30.8)
Gastro-intestinal, severe	5 (2.3)
Brain/nervous system	17 (7.9)
Treatment given^b^, no. (%)	
No treatment given	119 (55.6)
Antihistamine	64 (29.9)
Epinephrine administered	1 (0.5)
Unknown	31 (14.5)
Measure taken regarding OIT^b^, no. (%)	
No adjustment of therapy	134 (62.6)
Dose reduction	48 (22.4)
Clinical restart of OIT	7 (3.3)
OIT under antihistamine	25 (11.7)
Discontinued OIT	5 (2.3)
Emergency department visit, no. (%)	2 (0.9)

OIT, oral immunotherapy; t, OIT trajectories; mo, months.

a Allergic reactions were classified using Sampson's Severity Score.

b More than one option can be applicable.

No treatment was needed for 119 (55.6%) of 214 dosing reactions. Epinephrine was administered in one of 124 participants for a severe (Sampson III) at-home dosing reaction characterized by generalized urticaria, angio-edema, severe rhinitis, and repetitive vomiting. All Sampson IV reactions were only treated with a single dose of antihistamine, because parents observed only mild hoarseness or mild wheezing, without signs of dyspnea or severe illness. The single dose of antihistamine led to quick recovery. The four Sampson III reactions not treated with adrenaline were characterized by intermittent vomiting, with spontaneous clinical recovery.

In more than half of the cases, no adjustment of therapy was necessary after a dosing reaction (Table 2). When adjustments were needed, they included temporary dose reduction (48 cases, 22.4%), temporary interruption of therapy with clinical restart (7 cases, 3.3%), and temporary pretreatment with antihistamines (25 cases, 11.7%).

### Allergic dosing reactions categorized by baseline reaction threshold

Supplementary analyses were performed to compare the occurrence and characteristics of allergic dosing reactions between trajectories with a low baseline threshold (1–300 mg) and trajectories with a high baseline threshold (1,000–3,000 mg) (Supplementary Table S2). Allergic dosing reactions occured more frequently in OIT trajectories with a lower baseline threshold (68/100 trajectories, 68% vs. 42/88 trajectories, 47.7%). In both groups, the majority of reactions were mild (Sampson I–II). Severe reactions (Sampson III or higher) were rare, with no Sampson IV–V reactions observed in the high-threshold group. In both groups, the most commonly affected organ system was the skin and mucosa, followed by the gastrointestinal tract. In the lower-threshold group, 35.9% of allergic dosing reactions were treated with antihistamines, which was 19.0% in the higher-threshold group. Emergency department visits were infrequent and comparable between groups.

### Predictors of systemic allergic dosing reactions

Predictors of systemic dosing reactions (Sampson II-V) were assessed for the most frequently treated allergens in this study (Supplementary Tables S3). For peanut and hazelnut, children with systemic dosing reactions were more likely to have had a severe baseline allergic reaction compared to those who had either no or only localized dosing reactions (peanut 38.9% and hazelnut 44.4% vs. peanut 8.8% and hazelnut 8.3%, peanut *P* = 0.02; hazelnut *P* = 0.04) (Supplementary Tables S3a,c). Furthermore, for peanut, children with systemic dosing reactions more often had a low baseline reaction threshold (≤300 mg) than those without or with only localized dosing reactions, (72.2% vs. 29.4%, *P* = 0.01) (Supplementary Table S3a). Moreover, for cashew nut, gender was significantly different between participants with systemic dosing reactions and those without or with localized reactions, with men being more likely to have systemic dosing reactions (92.9% vs. 56.3%, *P* = 0.02) (Supplementary Table S3d). Age, single or multifood OIT, regular or rush dose escalation, and baseline level of sIgE were not predictors of systemic allergic dosing reactions.

### Non-allergic side effects

Non-allergic adverse events potentially related to OIT occurred in 32 (25.8%) of 124 participants (Table 3). A decrease in the occurrence of symptoms was seen during OIT. The most common symptom was flare-up of atopic dermatitis, reported by 27 (21.8%) of 124 participants. One participant who received hen's egg OIT was clinically diagnosed with eosinophilic gastrointestinal disease (persistent diarrhea, general malaise, and peripheral eosinophilia), which recovered within two months of discontinuing OIT.

**Table 3:** Non-allergic symptoms during OIT.

Characteristic	Total *n* = 124, *t* = 189
No. of patients with non-allergic symptoms, *n* (%)	32 (25.8)
No. of non-allergic AEs reported^a^, no. (%)	
Total	50 (100)
Dose escalation phase; first dose	0 (0)
Dose escalation phase; remaining	25 (50)
Maintenance phase; 0–4 mo	16 (32)
Maintenance phase; 4–8 mo	6 (12)
Maintenance phase; 8–12 mo	3 (6)
Type of non-allergic symptom^b^, *n* (%)	
Atopic dermatitis flare-up	27 (21.8)
Itching	4 (3.2)
Altered defecation pattern	6 (4.8)
Behavioral change	6 (4.8)
Tiredness	2 (1.6)
Measure taken regarding OIT^b^, no (%)	
No adjustment of therapy	35 (70)
Dose reduction	8 (16)
Clinical restart of OIT	0 (0)
OIT under antihistamine	5 (10)
Discontinued OIT	5 (10)

t, OIT trajectories; OIT, oral immunotherapy; mo, months.

a Symptoms may be reported more than once.

b More than one option can be applicable.

### Dropouts

Dropouts occurred in all study phases, but most frequently in the first half of the maintenance phase (Figure 2 and Table 4). The total number of dropouts was twelve participants and 27 OIT trajectories, of which ten (37%) trajectories dropped out because of therapy-related AEs (Supplementary Table S4a). These AEs were therapy resistant atopic dermatitis (t = 2) and persistent mild allergic side effects (t = 8) such as contact dermatitis, oral itching, and altered behavior. There were dropouts due to AEs for various allergens, with equal frequency during dose escalation and maintenance dosing, single and multifood OIT, and regular and rush dose escalation. Fourteen (51.9%) trajectories were discontinued because of aversion to the specific food, four of which also involved side effects of therapy (Supplementary Table S4b). Dropouts due to aversion occurred mainly in the maintenance phase, for different allergens, and for both single and multifood OIT. The age at initiation of OIT was higher in children who dropped out because of therapy-related AEs or aversion than in the entire study population (median 25.5 months, IQR 20.3–26 months, *P* < 0.01).

**Table 4:** Dropout during OIT.

Characteristic	Total *n* = 124, *t* = 189
No. of participants who dropped out, *n* (%)	12 (9.7)
No. of trajectories that dropped out, t (%)	27 (14.3)
Dropout allergen, no (%)	
Cow's milk	1 (3.7)
Henn's egg	4 (14.8)
Peanut	9 (33.3)
Walnut	3 (11.1)
Hazelnut	5 (18.5)
Cashew nut	4 (14.8)
Lentil	1 (3.7)
Almond	0 (0)
Pine nut	0 (0)
Wheat	0 (0)
Sesame	0 (0)
Dropout reason^a^, no (%)	
Adverse reactions	10 (37.0)
Aversion	14 (51.9)
Own initiative	7 (25.9)
Personal circumstances	6 (22.2)
Other health issues	2 (7.4)
Lost to follow-up	2 (7.4)
Dropout phase, no (%)	
Dose escalation	8 (29.6)
Maintenance 0–6 mo	10 (37.0)
Maintenance 6–12 mo	6 (22.2)
12 mo maintenance—exit OFC	3 (11.1)

OIT, oral immunotherapy; t, OIT trajectories; mo, months; OFC, oral food challenge.

a More than one option can be applicable.

## Discussion

This study aimed to assess the safety of one year of low-dose OIT in children under 24 months old, including those with multiple food allergies, using a newly developed feasibility-oriented OIT protocol. Important aspects of this OIT protocol include a personalized dose escalation schedule with as few hospital visits as possible, low-dose OIT, and simultaneous treatment of multiple food allergies. Overall, we found that OIT was safe, with almost all allergic reactions being mild and resolving spontaneously or with antihistamines. Severe reactions were rare, and there were no differences in reaction severity between single and multifood OIT, dose escalation strategies, or different allergens. While repeated side effects could be burdensome, and non-allergic side effects (e.g., flare-ups of atopic dermatitis) were common and let to therapy discontinuation in some cases, OIT was generally well tolerated.

Only a few studies have been published on the safety of preschool OIT (13–20, 24, 25). These studies had considerable heterogeneity in their study protocols. For instance, the protocols differ in terms of inclusion criteria, dose escalation schedules, maintenance doses, and duration of maintenance therapy. It is important to keep these differences in mind when comparing study outcomes. Despite the differences in study design, our study showed a side effect profile very similar to that of other preschool OIT studies. Many participants (71%–98%) experience AEs likely related to OIT, but these AEs are generally mild (85%–100%) (13–20, 24, 25). The most common AEs in the current study were mild gastrointestinal, localized skin, and upper airway symptoms, mirroring those documented in previous studies. Severe dosing reactions were rare in preschool OIT studies, with the use of epinephrine for dosing reactions being highly infrequent (≤3% of participants) (13, 17–19, 24, 25). However, in a randomized peanut OIT study, epinephrine was administered in 22% of participants (14). It is striking that, this study documented only five severe reactions (mainly laryngeal/throat symptoms or dyspnoea), indicating a low threshold for administering epinephrine. The safety of multifood OIT in preschoolers was assessed in one study and the results were similar to those of our study: all participants experienced dosing reactions, all of which were mild (19). Finally, the dropout rate due to AEs in our study aligned with that of previous studies on preschool peanut and tree nut OIT: 5% (10/189) in our study, and 3%–8% in previous studies (13, 19, 24). Taken together, our study showed that a feasibility-oriented OIT protocol does not affect safety.

This study has several strengths. First, this study focused on preschool-aged children with proven food allergies, which is a promising target group because in previous OIT studies many preschool-aged children achieved sustained unresponsiveness. Second, this study is unique in comprehensively investigating preschool OIT for various allergens and children with multiple food allergies. The OIT protocol was designed to be appropriate for all preschool-aged children with food allergy. In addition, the current protocol was designed to limit the impact of therapy on the child, parents, and health care system. Efforts to limit this impact included the use of commonly available food products, and reducing the number of hospital visits by rush dose escalation and a starting dose of 30% of the reaction threshold. Such a protocol is indispensable to ensure the feasibility of OIT within the existing health care infrastructure, especially if OIT becomes the standard of care in the future. Studying the safety of such a protocol, as we did in this study in a substantial group of children, is an important and pivotal step forward.

This study also has some limitations. First, the OIT protocol was optimized during the course of the study by adding rush dose escalation and simultaneous treatment of multiple food allergies, which introduced heterogeneity within the study population. However, this is inherent to the study design, namely an explorative intervention study, and provided the opportunity to compare safety outcomes between different treatment approaches, which offered valuable insights for future protocols and clinical practice. Another limitation is the small sample size of individual food allergens. While the current findings imply allergen-specific predictors of systemic allergic dosing reactions, the small sample size per allergen may have hindered the identification of clinically important predictors. It would be interesting to conduct future studies with sufficient power for different allergens, also to investigate whether the effectiveness of OIT differs between food allergens. Furthermore, a limitation of the current study is that parents were not asked to record the timing of AEs in a diary. Instead, AEs were reported retrospectively, which sometimes created uncertainty about the timing of AEs and, consequently, whether symptoms were attributable to the therapy. To address this limitation, a conservative approach was applied through worst-case documentation of AEs, which may have led to an overestimation of therapy-related AE frequency. In addition, the frequency of therapy-related AEs may be overestimated because no control group of standard care or placebo was included in this study. However, this overestimation is preferable in a safety analysis. Lastly, the Sampson severity grading system, though widely used and well-established for food-induced allergic reactions, may not capture the full spectrum of reaction severity. Alternative grading systems, like the CoFAR Grading Scale 3.0, could offer more precise assessments and improve study comparability.

## Conclusion

The ORKA study shows that OIT, performed according to a novel feasibility-oriented protocol, appears safe for preschoolers with various IgE-mediated food allergies and even multiple food allergies. Many children experience allergic dosing reactions, but most reactions are mild, and self-limiting or treatable with antihistamines. Although most children seem to experience little discomfort from side effects, they can be bothersome when they occur repeatedly and may lead to therapy withdrawal. These safety outcomes warrant further research into preschool OIT for common food allergens. The research group involved is currently further exploring the efficacy of this novel protocol in an ongoing multicenter randomized controlled trial (26). If ongoing and future studies confirm the efficacy of preschool OIT, it could significantly change the standard of care for food allergy. OIT may be a rescue for many preschool-aged children in whom food allergy has already reached clinical disease expression.

## Data Availability

The raw data supporting the conclusions of this article will be made available by the authors, without undue reservation.
