# Supplementary material for: Safety of a novel oral immunotherapy approach in preschool children with single and multiple food allergies
Source: Front Allergy. 2026 Feb 2;7:1724759. doi: 10.3389/falgy.2026.1724759 (PMC12907441; doi:10.3389/falgy.2026.1724759)
Supplement: Supplementary file 1 [file Datasheet1.docx]

**SUPPLEMENTARY INFORMATION: ORKA Study**

TABLE OF CONTENTS

1. Supplemental Methods………………………………………………………………….2-7
   1. Inclusion and Exclusion Criteria………………………………………………..2
   2. Oral Immunotherapy Protocol…………………………………………………..2-6
   3. Oral Food Challenge Protocol…………………………………………………..5-6
   4. Grading System for Allergic Reactions………………………………………...6
2. Supplemental Tables…………………………………………………………………...7-15
3. References……………………………………………………………………………….16
4. ***SUPPLEMENTAL METHODS***

**1a. INCLUSION AND EXCLUSION CRITERIA**

*Inclusion Criteria:*

Patients must meet *all* of the following criteria to be eligible for this study:

1. Age 9 months to less than 24 months, either gender.

2. Proven IgE-mediated food based on

a. Wheal ≥ 3mm on skin prick test to extract of the allergen concerned compared to a negative control, and/or serum IgE to the allergen concerned of > 0.35 kU/L.

AND

b. A systemic clinical reaction at ingestion of the allergen concerned during the screening oral food challenge.

3. The presence of other IgE mediated food allergies has been assessed by dietary introduction and/or sensitization testing. Of the food allergens listed under 2. dietary introduction has occured or a food allergy has been diagnosed.

4. Written informed consent from both parents/guardian.

*Exclusion Criteria:*

Patients who meet any of the following criteria will *not* be eligible for this study:

1. Uncontrolled asthma/viral wheezing defined as > one hospitalization for these symptoms in the past six months.

2. Uncontrolled atopic dermatitis.

3. Severe gastrointestinal symptoms such as gastroesophageal reflux disease in which an

underlying disease such as Eosinophilic Esophagitis (EoE) has not been ruled out.

4. Active eosinophilic gastrointestinal disease.

5. Mastocytosis including cutaneous mastocytosis.

6. Psychosocial problems in the family that may interfere with proper daily implementation of therapy.

7. Parental inability to follow instructions, recognize allergic reactions or administer emergency medication.

8. Participation in any interventional study during the ORKA Trial, except for studies on guided early

introduction of food allergens.

**1b. ORAL IMMUNOTHERAPY PROTOCOL**

*Food Products:*

All products that clearly state the protein content of the allergen concerned, for example:

Hen’s egg:

- Boiled egg, mashed
- Fried egg
- Home made pancake

Peanut:

- Peanut flour
- Peanut butter
- Unroasted and unsalted peanuts, ground

Cow’s milk:

- Semi-skimmed cow’s milk
- Yoghurt
- Custard

Cashew nut:

- Cashew nut flour
- Cashew nut butter
- Unroasted and unsalted cashew nuts, ground

Hazelnut:

- Hazelnut flour
- Hazelnut butter
- Unroasted and unsalted hazelnuts, ground

Walnut:

- Walnut flour
- Walnut butter
- Unroasted and unsalted walnuts, ground

*Study Phases:*

*Dose Escalation Phase:*

Oral immunotherapy (OIT) was started within two weeks to three months after the oral food challenge. To build up, increasing doses of the allergen were administered in the clinic in 1 to 14 visits to a maintenance dose of 300 mg of allergen protein with at least a 2-week interval between visits. Between clinic visits, patients ingested the same amount of food at home daily. Parents and the study team together chose for either a regular- or a rush schedule. In both schedules, a single dose of 30% of the threshold level (as determined during the oral food challenge) was given on the first day.

1. *Regular dose escalation schedule*

One dose increase was administered on a single day. A set schedule was followed as shown in the table below. The dose escalation phase consisted of one to fourteen build-up days, but could be extended if symptoms occurred during a build-up day or if a dose reduction was required (due to symptoms or missed doses). Children with multiple food allergies received OIT for all allergens eligible for inclusion, up to a maximum of four. For those with multiple food allergies, dose increases were performed on the same days.

| **Build-up day** | **Threshold level (mg of allergen protein)** | | | | | | |
| --- | --- | --- | --- | --- | --- | --- | --- |
|  | **3 mg** | **10 mg** | **30 mg** | **100 mg** | **300 mg** | **1000 mg** | **3000 mg** |
| **1** | 1 mg | 3.3 mg | 10 mg | 33 mg | 100 mg | 300 mg | 300 mg |
| **2** | 2 mg | 6.6 mg | 20 mg | 66 mg | 125 mg |  |  |
| **3** | 4 mg | 13.2 mg | 40 mg | 75 mg | 160 mg |  |  |
| **4** | 8 mg | 26.4 mg | 75 mg | 100 mg | 200 mg |  |  |
| **5** | 16 mg | 52.8 mg | 100 mg | 125 mg | 250 mg |  |  |
| **6** | 32 mg | 75 mg | 125 mg | 160 mg | 300 mg |  |  |
| **7** | 64 mg | 100 mg | 160 mg | 200 mg |  |  |  |
| **8** | 75 mg | 125 mg | 200 mg | 250 mg |  |  |  |
| **9** | 100 mg | 160 mg | 250 mg | 300 mg |  |  |  |
| **10** | 125 mg | 200 mg | 300 mg |  |  |  |  |
| **11** | 160 mg | 250 mg |  |  |  |  |  |
| **12** | 200 mg | 300 mg |  |  |  |  |  |
| **13** | 250 mg |  |  |  |  |  |  |
| **14** | 300 mg |  |  |  |  |  |  |

1. *Rush dose escalation schedule*

Starting on the second build-up day, two or three dose increases were administered in a day with at least 30 minutes between doses. The choice of a 2-step or 3-step schedule was made in consultation with parents. Children with multiple food allergies received OIT for all allergens eligible for inclusion, up to a maximum of four. For those with multiple food allergies, dose increases were performed on the same days, with a maximum total of six dose escalations on one day. The goal was to complete the dose escalation phase for all allergens simultaneously. For children on a rush schedule, the dose escalation phase consisted of one to eight build-up days, but could be extended if symptoms occurred during a build-up day or if a dose reduction was required (due to symptoms or missed doses).

| **Build-up day** | **Threshold level (mg of allergen protein)** | | | | | | | | | | | |
| --- | --- | --- | --- | --- | --- | --- | --- | --- | --- | --- | --- | --- |
|  | **3 mg** | | **10 mg** | | **30 mg** | | **100 mg** | | **300 mg** | | **1000 mg** | **3000 mg** |
|  | **3-step** | **2-step** | **3-step** | **2-step** | **3-step** | **2-step** | **3-step** | **2-step** | **3-step** | **2-step** | **1-step** | **1-step** |
| 1 | 1 mg | 1 mg | 3.3 mg | 3.3 mg | 10 mg | 10 mg | 33 mg | 33 mg | 100 mg | 100 mg | 300 mg | 300 mg |
| 2 | 2 mg | 2 mg | 5 mg | 5 mg | 15 mg | 20 mg | 50 mg | 50 mg | 150 mg | 125 mg |  |  |
|  | 3 mg | 3 mg | 8 mg | 8 mg | 22,5 mg | 30 mg | 75 mg | 75 mg | 225 mg | 150 mg |  |  |
|  | 4 mg |  | 12 mg |  | 33 mg |  | 100 mg |  | 300 mg |  |  |  |
| 3 | 6 mg | 4 mg | 18 mg | 18 mg | 50 mg | 45 mg | 150 mg | 100 mg |  | 225 mg |  |  |
|  | 10 mg | 6 mg | 27 mg | 27 mg | 75 mg | 67 mg | 225 mg | 150 mg |  | 300 mg |  |  |
|  | 15 mg |  | 40 mg |  | 100 mg |  | 300 mg |  |  |  |  |  |
| 4 | 25 mg | 10 mg | 60 mg | 40 mg | 150 mg | 100 mg |  | 225 mg |  |  |  |  |
|  | 37,5 mg | 15 mg | 90 mg | 60 mg | 225 mg | 150 mg |  | 300 mg |  |  |  |  |
|  | 60 mg |  | 135 mg |  | 300 mg |  |  |  |  |  |  |  |
| 5 | 90 mg | 25 mg | 175 mg | 90 mg |  | 225 mg |  |  |  |  |  |  |
|  | 135 mg | 37,5 mg | 250 mg | 135 mg |  | 300 mg |  |  |  |  |  |  |
|  | 175 mg |  | 300 mg |  |  |  |  |  |  |  |  |  |
| 6 | 250 mg | 60 mg |  | 175 mg |  |  |  |  |  |  |  |  |
|  | 300 mg | 90 mg |  | 250 mg |  |  |  |  |  |  |  |  |
| 7 |  | 135 mg |  | 300 mg |  |  |  |  |  |  |  |  |
|  |  | 175 mg |  |  |  |  |  |  |  |  |  |  |
| 8 |  | 250 mg |  |  |  |  |  |  |  |  |  |  |
|  |  | 300 mg |  |  |  |  |  |  |  |  |  |  |

*Maintenance Phase:*

Participants continued on daily OIT at a dose of 300 mg of allergen protein with a return visit after six months. The maintenance phase comprised 52 weeks.

*Avoidance Phase:*

At the end of the maintenance phase, participants stopped OIT and avoided consumption of the allergen concerned. At the completion of this phase, participants underwent an oral food challenge. The avoidance phase comprised 28 days (four weeks).

*Post-challenge:*

If participants did not have a clinical reaction during the oral food challenge at the end of the

avoidance phase, they were allowed to consume the allergen concerned. We recommended

offering a normal child portion (4.4 grams of allergen protein) at least weekly.


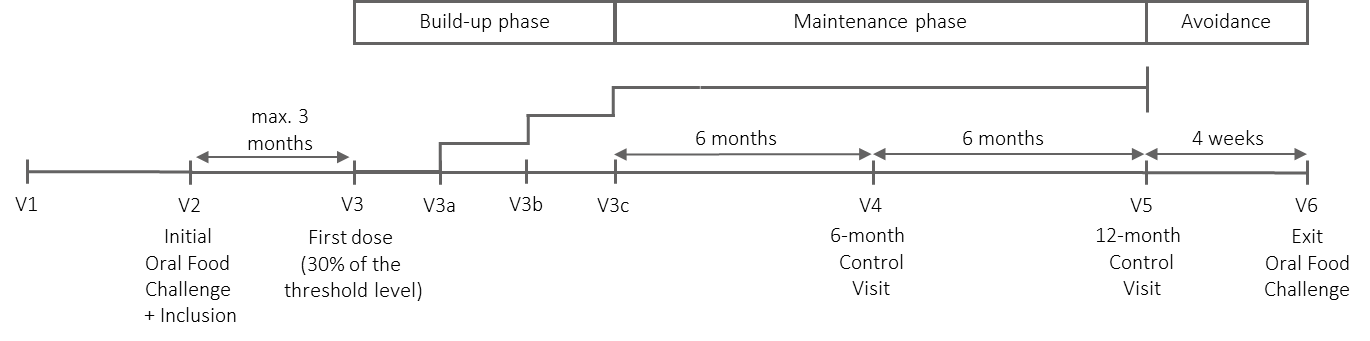
**Study Procedures**

***Missed doses for non-compliance***

Missed doses pose a potential risk to the study participant in all phases of the study. Therefore,

when doses were missed, a set algorithm was followed. Parents received instructions on this

algorithm and could read it back in a flow sheet for home use.

The algorithm for missed consecutive doses is as follows:

- 1 dose: the next dose would be the current dose and could be given at home.
- 2 doses in a row: the next dose would be 50% of the current dose and could be given at home.
- 3 or more doses in a row: the next dose would be 50% of the current dose and would be given under observation in the clinical research unit.

After a dose reduction, dose escalation would occur at home on a set schedule with an escalation no sooner than every three days with dose increases of one dose level at each escalation. If symptoms occurred, parents were asked to treat an allergic reaction according to the flow sheet (appendix 1) and contact the study team for instructions regarding dosing. The study team would contact the principal investigator for all missed doses due to dose-related symptoms and for three or more missed doses in a row.

***Management of dosing during concurrent illness***

If a participant had a mild cold or mild gastroenteritis, the parent was instructed to continue dosing on the current dose. If a participant had gastroenteritis with frequent vomiting without previous anaphylaxis (grade III-V), shortness of breath with wheezing, or fever above 39 °C and was markedly less active than normal, the parent was instructed to continue dosing at 50% of the current dose and apply the dose escalation algorithm on the flow sheet (appendix x). If a participant had gastroenteritis with frequent vomiting and previous anaphylaxis, parents were instructed to stop OIT administration and call the study team for instructions regarding dosing. Depending on the severity of the illness, the study team could instruct parents to hold dosing. Restart of dosing was done according to the dosing algorithm. In case of other illness or when in doubt, parents were asked to contact the study team for instructions on dosing.

***Documentation of dose-related symptoms***

Dosing was modified for dose-related symptoms, illness or other circumstances per protocol. All

adverse events, including dose-related symptoms, were captured in the patient record and reported in the electronic data capture system after contact with the study team. All adverse reactions that occurred during the dose escalation or maintenance phase were assumed to be dose-related unless there was a clear other explanation. Allergic dose-related symptoms were scored for severity by using a modified version of the Sampson grading system for allergic reactions[^1^](#_ENREF_1).

Flow sheet instructions for parents

Parents received a flow sheet with instructions for daily OIT dose administration, dealing with allergic reactions or missed doses, and dosing during concurrent illness. See appendix 1.

**1c. ORAL FOOD CHALLENGE PROTOCOL**

***Oral food challenges (4443 mg)***

Oral food challenges conducted in the study were open oral food challenges. However, the physician could use clinical judgement to choose for a double-blind placebo-controlled food challenge (DBPCFC). All oral food challenges were undertaken under direct medical supervision in the hospital, with emergency medication and staff immediately available. A standardized procedure was followed. Prior to an oral food challenge, children did not use antihistamines for three days and were assessed for fever, active wheezing, and a flare-up of atopic dermatitis. The oral food challenge consisted of seven gradually increasing doses at 30-minute intervals. However, based on clinical judgement, the physician could decide to increase the intervals between doses or repeat doses if there was concern that a reaction was developing. The set doses were 3, 10, 30, 100, 300, 1000, 3000 mg of allergen protein (cumulative dose of 4443 mg). A food challenge was performed on a single day, except for DBPCFC (on two days). Food challenges for different food allergens were performed on separate days.

***Oral food challenge outcome***

Regular checks were made for skin, gastrointestinal tract, and/or respiratory tract symptoms. The outcome of the oral food challenge was determined using the criteria in the table below (PRACTALL^1^). An oral food challenge was considered positive by the presence of one or more major criteria and/or three or more minor criteria. Otherwise, the oral food challenge was considered negative. An oral food challenge was also discontinued and considered positive if, in the physician’s judgment, the child had an allergic reaction even though scoring criteria were not met. All symptoms had to be of new onset, not due to pre-existing illness/condition, and occur within two hours of the last dose ingestion.

For positive challenges, the reaction threshold was defined as the last fully ingested dose after which the challenge was stopped.

| **Criteria for determining the outcome of an oral food challenge**^1^ |
| --- |
| **Major Criteria** |
| Confluent erythematous pruritic rash (>50%)  At least 3 urticarial lesions  At least 1 site of significant angioedema  Respiratory signs (at least one of the following):  Wheezing  Significant hoarseness  Frequent dry cough  Use of assistance breathing muscles  Continuous rubbing of nose or eyes  Long sneezes  Persistent rhinorrhoea  At least 2 distinct episodes of vomiting  Hypotension for age or loss of consciousness not associated with a vasovagal episode |
| **Minor Criteria (at least 3)** |
| Continuous, hard scratching  1-2 urticarial lesions  Mild angioedema of the lips  Multiple patches of erythematous rash which is not a flare-up of atopic dermatitis  Continuous complaint of throat tightness, pruritus and/or pain  At least 3 episodes of throat clearing  Intermittent rubbing of nose or eyes  Frequent nose picking  <10 intermittent sneezes  Single episode of vomiting  Notably distressed because of nausea and/or abdominal pain with decreased activity  Clear change in affect: whining, crying, and/or clinging to the parent |

**2d. Grading system for allergic reactions**

***Based on Sampson’s grading system***[***^1^***](#_ENREF_1)

|  | **Grade I** | **Grade II** | **Grade III** | **Grade IV** | **Grade V** |
| --- | --- | --- | --- | --- | --- |
|  | **Mild** | **Mild** | **Severe** | **Severe** | **Severe** |
| **Skin** | Localized prutitus, flushing, urticaria, angioedema | Generalized pruritus, flushing, urticaria, angioedema |  |  |  |
| **Gastrointestinal tract** | Oral pruritus, oral “tingling”, mild lip swelling | Nausea and/or emesis x’s 1 | Repetitive vomiting |  |  |
| **Upper airways** | X | Sneezing or mild rhinitis | Severe rhinitis, sensation of throat tightness | Stridor, swallowing problems, hoarseness |  |
| **Pulmonary** | X | X | X | Cough, wheezing or dyspnea | Respiratory arrest |
| **Cardiovascular** | X | X | Tachycardia | Mild hypotension or dysrhythmia | Bradycardia, circulatory shock |
| **Neurological** | X | Change in activity level | Anxiety | “Light headedness” feeling (presyncope) and anxiety | Loss of consciousness |

*Note: Not all symptoms need to be present. The severity score should be based on the organ system most affected.*

**Supplemental Tables**

**Table S1. Baseline characteristics grouped by reaction threshold**

|  | **Baseline threshold**  **1-300 mg** | **Baseline threshold 1000-3000 mg** |
| --- | --- | --- |
| **Characteristic** | **t=100** | **t= 88** |
| Age (mo) at start of OIT, median (IQR) | 20.5 (14.0-24.0) | 18.0 (14.0-23.0) |
| Grade of allergic reaction at entrance OFC † , t (%) |  |  |
| *Grade II* | 76 (76.0) | 75 (85.2) |
| *Grade III* | 13 (13.0) | 5 (5.7) |
| *Grade IV* | 10 (10.0) | 7 (8.0) |
| *Grade V* | 1 (1.0) | 1 (1.1) |
| *Median grade (IQR)* | 2 (2-2) | 2 (2-2) |
| Baseline sIgE (kU/L), median (IQR) |  |  |
| *Peanut* | 9.5 (3.5-15.0) | 5.5 (2.2-15.3) |
| *Ara H2 (peanut)* | 5.0 (2.06-13.5) | 3.0 (1.5-8.1) |
| *Hen's egg* | 19.0 (3.4-35.0) | 7.5 (2.8-15.0) |
| *Cow's milk* | 7.9 (2.8-87.3) | 25.0 (6.2-52.3) |
| *Hazelnut* | 12.7 (5.5-27.0) | 18.1 (11.6-23.0) |
| *Cor A9 (hazelnut)* | 2.7 (1.2-5.3) | 2.9 (1.5-3.8) |
| *Cor A14 (hazelnut)* | 10.4 (1.4-36.5) | 10.4 (0.7-28.0) |
| *Cashew nut* | 12.3 (7.5-31.7) | 8.4 (2.4-18.9) |
| *Walnut* | 10.2 (8.4-84.0) | 9.5 (6.6-52.3) |
| Food allergen treated with OIT, t (%) |  |  |
| *Peanut* | 24 (24.0) | 28 (31.8) |
| *Hen's egg* | 14 (14.0) | 23 (26.1) |
| *Cow's milk* | 5 (5.0) | 2 (2.3) |
| *Hazelnut* | 12 (12.0) | 9 (10.2) |
| *Cashew nut* | 29 (29.0) | 17 (19.3) |
| *Walnut* | 10 (10.0) | 6 (6.8) |
| *Almond* | 1 (1.0) | 1 (1.1) |
| *Pine nut* | 1 (1.0) | 1 (1.1) |
| *Sesame* | 2 (2.0 | 1 (1.1) |
| *Lentil* | 1 (1.0) | 0 (0) |
| *Wheat* | 1 (1.0) | 0 (0) |

*Abbreviations: OIT, Oral Immunotherapy; OFC, Oral Food Challenge; t, OIT trajectories; mo, months; IQR, interquartile range*

**Table S2. Allergic dosing reactions grouped by reaction threshold**

|  | **Baseline threshold**  **1-300 mg** | **Baseline threshold**  **1000-3000 mg** |
| --- | --- | --- |
|  | **t=100** | **t= 88** |
| No. of OIT trajectories with (an) allergic reaction(s), t (%) | 68 (68.0) | 42 (47.7) |
| No. of allergic reactions reported*, no. (%) | 167 (100.0) | 63 (100.0) |
| Severity †, no. (%) |  |  |
| *Grade I* | 76 (45.5) | 35 (55.6) |
| *Grade II* | 81 (48.5) | 26 (41.3) |
| *Grade III* | 5 (3.0) | 2 (3.2) |
| *Grade IV* | 5 (3.0) | 0 (0.0) |
| *Grade V* | 0 (0.0) | 0 (0.0) |
| Organ system involved ‡, no. (%) |  |  |
| *Lower airways* | 1 (0.6) | 0 (0.0) |
| *Upper airways, mild/moderate* | 38 (22.8) | 10 (15.9) |
| *Upper airways, severe* | 4 (2.4) | 0 (0.0) |
| *Cardiovascular system* | 0 (0.0) | 0 (0.0) |
| *Skin/mucosa, mild/moderate* | 94 (56.3) | 38 (60.3) |
| *Skin/mucosa, severe* | 31 (18.6) | 10 (15.9) |
| *Gastro-intestinal, mild/moderate* | 58 (34.7) | 15 (23.8) |
| *Gastro-intestinal, severe* | 5 (3.0) | 2 (3.2) |
| *Brain/nervous system* | 14 (8.4) | 4 (6.3) |
| Treatment given ‡, no. (%) |  |  |
| *No treatment given* | 87 (52.1) | 39 (61.9) |
| *Antihistamine* | 60 (35.9) | 12 (19.0) |
| *Epinephrine administered* | 2 (1.2) | 1 (1.6) |
| *Unknown* | 19 (11.4) | 14 (22.2) |
| Measure taken regarding OIT ‡, no. (%) |  |  |
| *No adjustment of therapy* | 105 (62.9) | 40 (63.5) |
| *Dose reduction* | 37 (22.2) | 16 (25.4) |
| *Clinical restart of OIT* | 4 (2.4) | 3 (4.8) |
| *OIT under antihistamine* | 22 (13.2) | 4 (6.3) |
| *Discontinued OIT* | 4 (2.4) | 1 (1.6) |
| Emergency Department visit, no. (%) | 3 (1.8) | 2 (3.2) |

*Note: *Dosing reactions may occur simultaneously in both groups if the reaction occurred after ingestion of multiple allergens; † allergic reactions were classified using Sampson's Severity Score; ‡ more than one option can be applicable*

*Abbreviations: OIT, Oral Immunotherapy; t, OIT trajectories; mo, months*

**Table S3a. Predictors of systemic dosing reactions during Peanut OIT**

| **Peanut t=52** | **No or grade I reaction(s)** | **Grade II-V allergic reaction(s)** | | ***P*-value** |
| --- | --- | --- | --- | --- |
|  | **t=34 (65%)** | **t=18 (35%)** | |  |
| Gender: male, t (%) | 22 (64.7) | 12 (66.7) | | 0.89* |
| Age (mo) at start of OIT, mean (SD) | 16.1 (5.7) | 19.2 (6.0) | | 0.07**** |
| Single or multiple OIT, t (%) |  |  | | 0.18* |
| *Single OIT* | 16 (47.1) | 12 (66.7) | |  |
| *Multiple OIT* | 18 (52.9) | 6 (33.3) | |  |
| Baseline threshold level, t (%) |  |  | | 0.01** |
| *1 mg* | 0 (0) | 0 (0) | |  |
| *3 mg* | 1 (2.9) | 0 (0) | |  |
| *10 mg* | 0 (0) | 0 (0) | |  |
| *30 mg* | 0 (0) | 2 (11.1) | |  |
| *100 mg* | 4 (11.8) | 2 (11.1) | |  |
| *300 mg* | 5 (14.7) | 9 (50) | |  |
| *1000 mg* | 11 (32.4) | 4 (22.2) | |  |
| *3000 mg* | 13 (38.2) | 1 (5.6) | |  |
| Baseline severity *†*, t (%) |  |  | | 0.02** |
| *Grade II* | 31 (91.2) | 11 (61.1) | |  |
| *Grade III* | 1 (2.9) | 4 (22.2) | |  |
| *Grade IV* | 2 (5.9) | 3 (16.7) | |  |
| *Grade V* | 0 (0) | 0 (0) | |  |
| Build-up schedule, t (%) |  |  | | 0.053* |
| *Regular* | 26 (76.5) | 9 (50) | |  |
| *Rush* | 8 (23.5) | 9 (50) | |  |
| Baseline sIgE pinda (kU/L), median (IQR) | 6.7 (2.1-15.3) | 9.3 (3-17) | | 0.68*** |
| Baseline sIgE Ara H2 (kU/L), median (IQR) | 4.7 (1.8-12) | 6.8 (2.5-14) | | 0.34*** |
|  |  |  | |  |
| **Table S3b. Predictors of systemic dosing reactions during Hen’s egg OIT** | | | |  |
| **Hen's egg t=38** | **No or grade I reaction(s)** | **Grade II-V allergic reaction(s)** | | ***P*-value** |
|  | **t=23 (61%)** | **t=15 (39%)** | |  |
| Gender: male, t (%) | 19 (82.6) | 10 (66.7) | | 0.44** |
| Age (mo) at start of OIT, median (IQR) | 17 (13-20) | 19 (13-23) | | 0.44*** |
| Single or multiple OIT, t (%) |  |  | | 0.96* |
| *Single OIT* | 14 (60.9) | 9 (60) | |  |
| *Multiple OIT* | 9 (39.1) | 6 (40) | |  |
| Baseline threshold level, t (%) |  |  | | 0.10** |
| *1 mg* | 0 (0) | 0 (0) | |  |
| *3 mg* | 0 (0) | 0 (0) | |  |
| *10 mg* | 0 (0) | 0 (0) | |  |
| *30 mg* | 0 (0) | 0 (0) | |  |
| *100 mg* | 3 (13) | 3 (20) | |  |
| *300 mg* | 5 (21.7) | 5 (33.3) | |  |
| *1000 mg* | 10 (43.5) | 1 (6.7) | |  |
| *3000 mg* | 5 (21.7) | 6 (40) | |  |
| Baseline severity *†*, t (%) |  |  | | 1.00** |
| *Grade II* | 20 (87) | 14 (93.3) | |  |
| *Grade III* | 2 (8.7) | 1 (6.7) | |  |
| *Grade IV* | 1 (4.3) | 0 (0) | |  |
| *Grade V* | 0 (0) | 0 (0) | |  |
| Build-up schedule, t (%) |  |  | | 0.19* |
| *Regular* | 17 (73.9) | 8 (53.3) | |  |
| *Rush* | 6 (26.1) | 7 (46.7) | |  |
| Baseline sIgE hen's egg (kU/L), median (IQR) | 7.5 (2.3-20) | 3.5 (2.6-19) | | 0.95*** |
|  |  |  | |  |
| **Table S3c. Predictors of systemic dosing reactions during Hazelnut OIT** | | | | |
| **Hazelnut t=21** | **No or grade I reaction(s)** | **Grade II-V allergic reaction(s)** | | ***P*-value** |
|  | **t=12 (57%)** | **t=9 (43%)** | |  |
| Gender: male, t (%) | 9 (75) | 5 (55.6) | | 0.64** |
| Age (mo) at start of OIT, mean (SD) | 21.3 (7.2) | 23.8 (3.6) | | 0.36**** |
| Single or multiple OIT, t (%) |  |  | | 1.00** |
| *Single OIT* | 3 (25) | 2 (22.2) | |  |
| *Multiple OIT* | 9 (75) | 7 (77.8) | |  |
| Baseline threshold level, median (IQR) |  |  | | 0.72** |
| *1 mg* | 0 (0) | 0 (0) | |  |
| *3 mg* | 0 (0) | 1 (11.1) | |  |
| *10 mg* | 0 (0) | 0 (0) | |  |
| *30 mg* | 1 (8.3) | 0 (0) | |  |
| *100 mg* | 1 (8.3) | 1 (11.1) | |  |
| *300 mg* | 5 (41.7) | 3 (33.3) | |  |
| *1000 mg* | 3 (25) | 4 (44.4) | |  |
| *3000 mg* | 2 (16.7) | 0 (0) | |  |
| Baseline severity *†*, t (%) |  |  | | 0.04** |
| *Grade II* | 11 (91.7) | 5 (55.6) | |  |
| *Grade III* | 0 (0) | 1 (11.1) | |  |
| *Grade IV* | 0 (0) | 3 (33.3) | |  |
| *Grade V* | 1 (8.3) | 0 (0) | |  |
| Build-up schedule, t (%) |  |  | | 0.64** |
| *Regular* | 5 (41.7) | 2 (22.2) | |  |
| *Rush* | 7 (58.3) | 7 (77.8) | |  |
| Baseline sIgE hazelnut (kU/L), median (IQR) | 18.7 (12.6-62) | 11.3 (4.2-24) | | 0.19*** |
| Baseline sIgE cor a14 (kU/L), median (IQR) | 18 (7.2-29.8) | 1.6 (0.7-44) | | 0.38*** |
|  |  |  | |  |
| **Table S3d. Predictors of systemic dosing reactions during Cashew nut OIT** | | | | |
| **Cashew nut t=46** | **No or grade I reaction(s)** | **Grade II-V allergic reaction(s)** | | ***P*-value** |
|  | **t=32 (70%)** | **t=14 (30%)** | |  |
| Gender: male, t (%) | 18 (56.3) | 13 (92.9) | | 0.02** |
| Age (mo) at start of OIT, median (IQR) | 19.5 (13-23) | 22 (16-26.3) | | 0.14*** |
| Single or multiple OIT, t (%) |  |  | | 0.75* |
| *Single OIT* | 13 (40.6) | 5 (35.7) | |  |
| *Multiple OIT* | 19 (59.4) | 9 (64.3) | |  |
| Baseline threshold level, median (IQR) |  |  | | 0.57** |
| *1 mg* | 0 (0) | 0 (0) | |  |
| *3 mg* | 0 (0) | 0 (0) | |  |
| *10 mg* | 4 (12.5) | 2 (14.3) | |  |
| *30 mg* | 3 (9.4) | 0 (0) | |  |
| *100 mg* | 6 (18.8) | 3 (21.4) | |  |
| *300 mg* | 6 (18.8) | 6 (42.9) | |  |
| *1000 mg* | 8 (25) | 2 (14.3) | |  |
| *3000 mg* | 5 (15.6) | 1 (7.1) | |  |
| Baseline severity *†*, t (%) |  |  | | 0.27** |
| *Grade II* | 26 (1.3) | 11 (78.6) | |  |
| *Grade III* | 5 (15.6) | 1 (7.1) | |  |
| *Grade IV* | 1 (3.1) | 2 (14.3) | |  |
| *Grade V* | 0 (0) | 0 (0) | |  |
| Build-up schedule, t (%) |  |  | | 0.61* |
| *Regular* | 14 (43.8) | 5 (35.7) | |  |
| *Rush* | 18 (56.3) | 9 (64.3) | |  |
| Baseline sIgE (kU/L), median (IQR) | 8.3 (2.6-22.3) | 12.6 (8.3-51.3) | | 0.06*** |
|  |  |  | |  |
| **Table S3e. Predictors of systemic dosing reactions during Walnut OIT** | | | |  |
| **Walnut n=16** | **No or grade I reaction(s)** | **Grade II-V allergic reaction(s)** | | ***P*-value** |
|  | **t=9 (56%)** | **t=7 (44%)** | |  |
| Gender: male, t (%) | 5 (55.6) | 6 (85.7) | | 0.31** |
| Age (mo) at start of OIT, mean (SD) | 22 (7.4) | 25 (2.3) | | 0.32**** |
| Single or multiple OIT, t (%) |  |  | | 0.32** |
| *Single OIT* | 6 (66.7) | 2 (28.6) | |  |
| *Multiple OIT* | 3 (33.3) | 5 (71.4) | |  |
| Baseline threshold level, median (IQR) |  |  | | 0.50** |
| *1 mg* | 0 (0) | 0 (0) | |  |
| *3 mg* | 0 (0) | 0 (0) | |  |
| *10 mg* | 1 (11.1) | 0 (0) | |  |
| *30 mg* | 0 (0) | 0 (0) | |  |
| *100 mg* | 0 (0) | 0 (0) | |  |
| *300 mg* | 3 (33.3) | 5 (71.4) | |  |
| *1000 mg* | 3 (33.3) | 2 (28.6) | |  |
| *3000 mg* | 2 (22.2) | 0 (0) | |  |
| Baseline severity *†*, t (%) |  |  | | 1.00** |
| *Grade II* | 6 (66.7) | 6 (85.7) | |  |
| *Grade III* | 1 (11.1) | 0 (0) | |  |
| *Grade IV* | 2 (22.2) | 1 (14.3) | |  |
| *Grade V* | 0 (0) | 0 (0) | |  |
| Build-up schedule, t (%) |  |  | | 0.15** |
| *Regular* | 5 (55.6) | 1 (14.3) | |  |
| *Rush* | 4 (44.4) | 6 (85.7) | |  |
| Baseline sIgE (kU/L), median (IQR) | 9.9 (3.3-17.1) | 11.4 (9.2-84) | | 0.40*** |
| *Note:* * *Chi-square test;* ** *Fisher's exact test;* *** *Mann-Whitney U test; ***** *independent t-test; † allergic reactions were classified using Sampson's Severity Score* | | |  |  |
| *Abbreviations: OIT, Oral Immunotherapy; t, OIT trajectories; IQR, interquartile range* | | |  |  |

| **Table S4a Dropout due to adverse events** | | | | |  |  |  |
| --- | --- | --- | --- | --- | --- | --- | --- |
| **Patient** | **Allergen** | **Age (mo) at start of OIT** | **Dose escalation** | **Single or multiple OIT** | **Adverse event(s) description** | **Measures taken regarding dosing** | **Dropout phase** |
| **1** | Walnut | 21 | Regular | Multiple | Therapy resistant atopic dermatitis | OIT dose reduction | Maintenance 6-12 mo |
| **2** | Hen's egg | 23 | Regular | Single | Therapy resistant atopic dermatitis, altered behavior | Antihistamine maintenance therapy, OIT dose reduction | Maintenance 6-12 mo |
| **3** | Peanut | 34 | Regular | Single | Contact dermatitis | None, parents had already quit OIT themselves | Maintenance 0-6 mo |
| **4** | Cashew nut | 26 | Rush | Multiple | Oral itching, contact dermatitis, single vomiting | None, parents had already quit OIT themselves | Dose escalation |
| **4** | Peanut | 26 | Rush | Multiple | Oral itching, contact dermatitis, single vomiting, mild rhinitis, flare up of atopic dermatitis | OIT dose reduction, antihistamine maintenance therapy | Dose escalation |
| **5** | Cashew nut | 25 | Regular | Multiple | Itching, contact dermatitis, flare up of atopic dermatitis | Antihistamine maintenance therapy | Maintenance 0-6 mo |
| **6** | Hazelnut | 18 | Rush | Multiple | Contact dermatitis, flare up of atopic dermatitis, mild rhinoconjunctivitis, altered behavior | OIT dose reduction, antihistamine maintenance therapy | Dose escalation |
| **7** | Lentil | 26 | Rush | Single | Single vomiting, mild angio-edema, <3 urticaria | OIT dose reduction | Dose escalation |
| **8** | Hazelnut | 17 | Regular | Single | Altered behavior, flare up of atopic dermatitis, oral itching | None, parents had already quit OIT themselves | Maintenance 0-6 mo |
| **9** | Peanut | 12 | Rush | Single | Contact dermatitis, flare up of atopic dermatitis | OIT dose reduction, antihistamine maintenance therapy | Dose escalation |

*Abbreviations: OIT, Oral Immunotherapy; mo, months*

| **Table S4b Dropout due to aversion** | | | | |  |  |
| --- | --- | --- | --- | --- | --- | --- |
| **Patient** | **Allergen** | **Age (mo) at start of OIT** | **Dose escalation** | **Single or multiple OIT** | **Measures taken regarding dosing** | **Dropout phase** |
| **1** | Hen's egg | 41 | Rush | Single | None, parents had already quit OIT themselves | Maintenance 0-6 mo |
| **2** | Hazelnut | 26 | Rush | Single | None, parents had already quit OIT themselves | Maintenance 0-6 mo |
| **3** | Cashew nut | 25 | Rush | Multiple | Other product | Maintenance 0-6 mo |
| **4** | Peanut | 20 | Regular | Single | Other product, other matrix | Maintenance 0-6 mo |
| **5** | Cashew nut | 25 | Regular | Multiple | None, parents had already quit OIT themselves | Maintenance 0-6 mo |
| **6** | Hazelnut | 17 | Regular | Single | Other product | Maintenance 0-6 mo |
| **7** | Hen's egg | 24 | Regular | Multiple | Other product, other matrix | Maintenance 6-12 mo |
| **8** | Peanut | 18 | Rush | Single | Temporary dose reduction, other product, other matrix | Dose escalation |
| **9** | Hen's egg | 28 | Rush | Multiple | Other product, other matrix | Maintenance 0-6 mo |
| **9** | Cashew nut | 28 | Rush | Multiple | Oher matrix | Dose escalation |
| **10** | Peanut | 26 | Rush | Multiple | Other matrix, other moment of dosing | Dose escalation |
| **10** | Cashew nut | 26 | Rush | Multiple | Other matrix, other moment of dosing | Dose escalation |
| **11** | Peanut | 26 | Regular | Multiple | None, parents had already quit OIT themselves | Maintenance 0-6 mo |
| **11** | Hazelnut | 26 | Regular | Multiple | Other product | Maintenance 0-6 mo |

*Abbreviations: OIT, Oral Immunotherapy; mo, months*

**References**

1. Sampson HA, Gerth van Wijk R, Bindslev-Jensen C, et al. Standardizing double-blind, placebo-controlled oral food challenges: American Academy of Allergy, Asthma &amp; Immunology–European Academy of Allergy and Clinical Immunology PRACTALL consensus report. *Journal of Allergy and Clinical Immunology*. 2012;130(6):1260-1274. doi:10.1016/j.jaci.2012.10.017
